# Oxytocin Elicits Itch Scratching Behavior via Spinal GRP/GRPR System

**DOI:** 10.3389/fnins.2020.581977

**Published:** 2020-09-23

**Authors:** Jing Guo, Xiyuan Ba, Megumi Matsuda, Pengfei Wei, Changyu Jiang, Wuping Sun, Lizu Xiao, Donglin Xiong, Xiang Liao, Yue Hao

**Affiliations:** ^1^Department of Endocrinology and Metabolism, Shenzhen University General Hospital, Shenzhen, China; ^2^Department of Pain Medicine and Shenzhen Municipal Key Laboratory for Pain Medicine, Shenzhen Nanshan People’s Hospital and the 6th Affiliated Hospital of Shenzhen University Health Science Center, Shenzhen, China; ^3^School of Pharmaceutical Science, Health Science Center, Shenzhen University, Shenzhen, China; ^4^Research Unit for the Neurobiology of Pain, Department of Anesthesiology, Kyoto Prefectural University of Medicine, Kyoto, Japan

**Keywords:** oxytocin, hindpaw scratching, itch, oxytocin receptor, gastrin-releasing peptide, spinal dorsal horn

## Abstract

Oxytocin (OT), a neuropeptide involved in the regulation of complex social and sexual behavior in mammals, has been proposed as a treatment for a number of psychiatric disorders including pain. It has been well documented that central administration of OT elicits strong scratching and grooming behaviors in rodents. However, these behaviors were only described as symptoms, few studies have investigated their underlying neural mechanisms. Thus, we readdressed this question and undertook an analysis of spinal circuits underlying OT-induced scratching behavior in the present study. We demonstrated that intrathecal OT induced robust but transient hindpaw scratching behaviors by activating spinal OT receptors (OTRs). Combining the pre-clinical and clinical evidence, we speculated that OT-induced scratching may be an itch symptom. Further RNAscope studies revealed that near 80% spinal GRP neurons expressed OTRs. OT activated the expression of *c-fos* mRNA in spinal GRP neurons. Chemical ablation of GRPR neurons significantly reduced intrathecal OT-induced scratching behaviors. Given GRP/GRPR pathway plays an important role in spinal itch transmission, we proposed that OT binds to the OTRs expressed on the GRP neurons, and activates GRP/GRPR pathway to trigger itch-scratching behaviors in mice. These findings provide novel evidence relevant for advancing understanding of OT-induced behavioral changes, which will be important for the development of OT-based drugs to treat a variety of psychiatric disorders.

## Introduction

Oxytocin (OT) is a 9-amino acid neuropeptide synthesized in the paraventricular and supraoptic nuclei of the hypothalamus (Sawchenko et al., 1983; Burbach et al., 2001). It is either transported to the posterior pituitary and secreted into the bloodstream to exert a variety of hormonal effects, or released into the central nervous system and acts as modulator of neuronal transmission (Brooks and Tracey, 2005; Kiss and Mikkelsen, 2005; Knobloch et al., 2012). OT has a central role in regulation of complex social and sexual behavior in mammals (Hurlemann et al., 2010; Meyer-Lindenberg et al., 2011; Wittig et al., 2014; Yoshihara et al., 2018) and has demonstrated the analgesic effects in both humans and rodents (Honda and Takano, 2009; Koshimizu and Tsujimoto, 2009). Due to the unique and multidimensional role in a wide range of behaviors, OT has become a promising target for therapeutic interventions in different fields (Goodin et al., 2015; Li et al., 2016). OT plays its effects mainly by activating OT receptors (OTRs), which belongs to the G protein-coupled receptor superfamily. OTRs and three structurally related arginine-vasopressin (AVP) receptors (V1aR, V1bR and V2R) form a receptor sub-family. All of these receptors bind to OT with different affinities and elicit different responses (Meyer-Lindenberg et al., 2011).

It has been well documented that central administration of OT elicits strong scratching and grooming behaviors in rats (Van Wimersma Greidanus et al., 1990; Yaksh et al., 2014) and mice (Meisenberg and Simmons, 1982; Thurston, 1992; Schorscher-Petcu et al., 2010). When injected into the brain or spinal cord, OT at a dose of 1 nmol caused a behavioral syndrome characterized by grooming, moving, foraging, and squeaking in mice or rats (Delanoy et al., 1978; Yaksh et al., 2014), whereas a lower dose (<0.1 nmol) of OT produced scratching, rather than grooming, as the predominant symptom of behavioral arousal in mice (Meisenberg and Simmons, 1982; Schorscher-Petcu et al., 2010). We also found that intrathecal OT, within a certain dose range, caused significant hindpaw scratching behavior in mice. However, central OT induced hindpaw scratching was often described as a behavioral symptom, few studies have investigated its underlying neural mechanisms.

Although it is not clear whether intrathecal OT elicits itch sensation, this hind limb scratching behavior is a typical itch symptom in rodents, and has been used increasingly to assess itch in animal models (Sun and Chen, 2007; Liu et al., 2009; Ross et al., 2010; Han et al., 2018; Wang et al., 2019). Recently, Li et al. reported that intradermal injection of OT aggravated chloroquine-induced itch responses (Li et al., 2019), combining with the clinical evidence that patients who received OT showed a risk of experiencing pruritus (Boucher et al., 2004; Rath, 2009), we hypothesized that intrathecal OT induced hindpaw scratching in mice is an itch symptom. Acute itch is very similar to pain, it serves as a protective mechanism that makes us response to potentially harmful stimuli or events. It has been found that gastrin-releasing peptide (GRP) and its receptor play a critical role in itch processing. Ablation of GRPR neurons in the spinal dorsal horn abolishes multiple pruritic stimuli induced scratching without affecting normal pain behavior (Sun and Chen, 2007; Sun et al., 2009). In the present study, we readdressed intrathecal OT induced hindpaw scratching behavior, and the role of spinal GRP/GRPR neurons in OT-induced scratching was also investigated to identify the spinal transmission neurons underlying this behavior.

## Materials and Methods

### Animals

Male and female C57BL/6 mice (starting weight of 18–22 g) were purchased from Guangdong province Laboratory Animal Center (Guangzhou, China). The mice were housed in plastic cages (5 per cage) in a temperature-controlled environment on a 12 h/12 h light/dark cycle. Food and water were available *ad libitum*. All animal procedures were conducted in strict adherence to the guidelines of the International Association for the Study of Pain and were approved by the Animal Care and Use Committee of Health Science Center at Shenzhen University. The animals were randomly assigned to the study groups and all behavioral testing was done with the experimenters blinded to the treatment conditions.

### Reagents

Oxytocin, TGOT, and dVOT were purchased from Bachem AG (Bubendorf, Switzerland). Atosiban and TC OT 39 were obtained from Tocris (MS, United States). RG7314 was purchased from MedChemExpress (NJ, United States). Morphine hydrochloride was produced by Shenyang First Pharmaceutical Factory (Shenyang, China). Bombesin-saporin was purchased from Wako (Osaka, Japan).

### Drug Injection

For intrathecal injection, spinal cord puncture was made by a Hamilton microsyringe (Hamilton) with a 30 G needle between the L5 and L6 level to deliver reagents (5 μl) to the cerebral spinal fluid. OT, OTR selective agonist TGOT or TCOT39 was given intrathecally. The behaviors of mice were then video recorded for 30 min immediately after drug injection. OTR or vasopressin 1a receptor (V1aR) selective antagonist was given 15 min before OT injection. For intradermal injection, OT (0.03 or 3 nmol in 50 μl saline) was injected into the loose skin on the back of the neck with a 30-G needle. We chose the dose of the reagents based on our previous reports (Sun et al., 2018), as well as our pilot study.

### Scratching Behavior Testing

The mice were habituated to experimenter handling briefly, and then placed in the behavioral testing apparatus for three to five ‘habituation’ sessions. On the testing day, the mice were habituated in the behavioral testing apparatus for 15 min. Their behaviors were then video recorded for 30 min after drug injection. The video was subsequently played back and the number of scratches by each mouse was quantified in a blinded manner. A scratch was counted when a mouse lifted its hind paw to scratch the back (the area of the back near abdomen). Room temperature and humidity levels remained constant and stable for all experiments.

### *In situ* Hybridization

*In situ* Hybridization procedures were performed to detect mRNA expression as described previously (Wang et al., 2020). The mice were deeply anesthetized with isoflurane and transcardially perfused with PBS followed by 4% PFA/1.5% picric acid. Following perfusion, lumbar spinal cord L3-5 were removed and immersed into PFA for 2 h at room temperature. The tissues were washed 3 times in PBS, followed by cryopreservation in 20% sucrose overnight followed by 30% sucrose overnight. Spinal cords were then embedded in optimal cutting temperature (OCT) medium (Tissue-Tek) and cryosectioned to produce 14 μm sections which were mounted onto Superfrost Plus charged slides. *In situ* hybridization was performed using the RNAscope system (Advanced Cell Diagnostics) following the manufacturer’s protocol and our previous reports (Wang et al., 2020). Pre-treatment consisted of dehydration, followed by incubation with hydrogen peroxide and protease IV at room temperature. The Multiplex Fluorescent Kit v2 protocol was followed using commercial probes for *Otr* (Mm-Oprm1-C3,NM_001081147.1, # 315841-C3), *Grp* (Mm-Grp-C1, NM_175012.4, # 317861), Grpr (Mm-Grpr-C1, NM_008177.2,#317871) and c-fos (Mm-Fos,NM_010234.2,#14281-C3). Visualized cells with more than 5 puncta per cell were classified as positive neurons.

### Spinal GRPR Neuron Ablation

The mice were given a single intrathecal injection of Bombesin-saporin (400 ng in 10 mL sterile saline, Advanced targeting Systems, San Diego, CA) to selective ablate spinal GRPR neurons (Sun et al., 2009). Most of GRPR neurons were lost two weeks after single intrathecal injection. Scratching behavioral testing was then conducted.

### Statistical Analysis

Data were expressed as the means ± SEM and analyzed by analysis of variance (ANOVA) using one-way or mixed factorial designs as appropriate, followed by Bonferroni’s *post hoc* test or simple-effects ANOVA. All statistical analysis was performed using Prim GraphPad 8.0. Significance was defined as *p* < 0.05.

## Results

### Intrathecal OT Induced Significant Hindpaw Scratching Behavior in Mice

**FIGURE 1:**
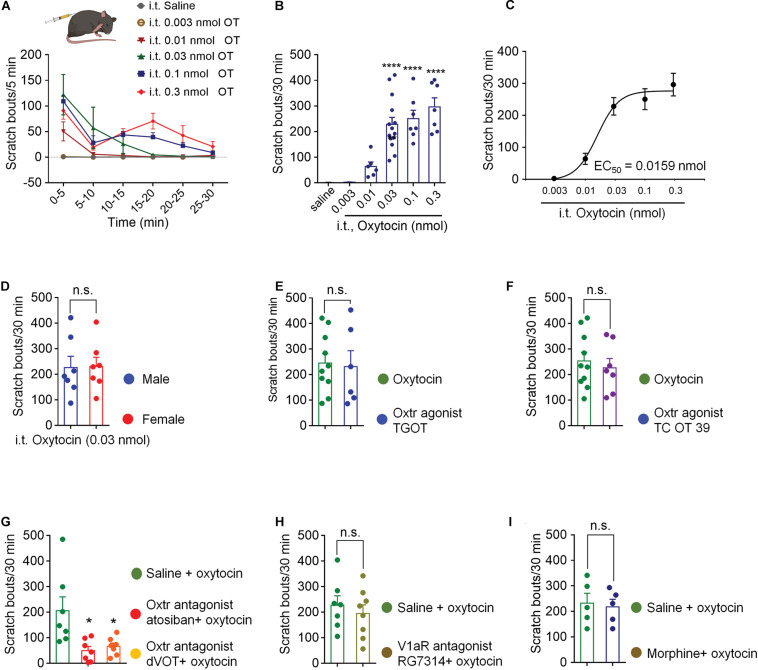
Intrathecal OT induced hindpaw scratching behavior was mediated by spinal OTRs. **(A)** Time course of different doses of intrathecal OT evoked scratching bouts in mice. OT at dose of 0.003, 0.01, 0.03, 0.1, and 0.3 nmol was administered intrathecally, scratching bouts of the mice were then recorded every 5 min for 30 min. **(B)** Different doses of intrathecal OT-induced scratching bouts in 30 min after injection. Data are expressed as mean ± SEM. **p < 0.01, ****p < 0.0001 vs. Saline; One-way ANOVA followed by Bonferroni’s *post hoc* test. **(C)** Dose-response curve of intrathecal OT-induced scratching behaviors. **(D)** Intrathecal OT-induced itch behavior in male and female mice. OT at dose of 0.03 nmol was administered intrathecally, scratching bouts was then recorded for 30 min. Data are expressed as mean ± SEM paired *T*- test. **(E)** Intrathecal administration of a selective OT receptor agonist, TGOT (0.03 nmol) caused scratching behaviors similar to OT in mice. **(F)** Intrathecal administration of selective V1aR antagonist, also a selective OT receptor agonist, TC OT 39 (0.1 nmol) caused significant scratching behaviors in mice. **(G)** Intrathecal administration of selective OT receptor antagonists, Atosiban (0.1 nmol) and dVOT (0.1 nmol) decreased OT induced scratching behaviors in mice. **(H)** Selective blockade of V1aR by pretreatment of its antagonist, RG7314 (0.1 nmol) did not prevent OT-induced scratching behaviors in mice. I Morphine (0.3 nmol, i.t.) had no effect on OT-induced scratching. Data are expressed as mean ± SEM paired *T*- test. *p < 0.05.

Intrathecal injection of OT evoked significant hindpaw scratching bouts in a dose-dependent manner. Figure 1A shows the time course of different doses of OT-induced scratching behaviors. OT-induced scratching behavior occurred immediately after injection, most frequently happened during the first 5 min with as many as 120 bouts, and then rapidly decreased by almost 50% in the next 5 min. Increase in dosage led to more bouts and a longer duration of the scratching behavior. Lower dose of OT (0.01 nmol, i.t.) induced scratching behavior only lasted 5 min, while higher dose of OT lasted 30 min. The total amount of OT-induced scratching bouts dose-dependently increased within 30-min (Figure 1B). Intrathecal OT at a dose as small as 0.01 nmol evoked significant scratching behaviors compared to the vehicle (*p* < 0.01; Figure 1B). Oxytocin at dose of 0.3 nmol evoked as many as 300 bouts within 30 min after injection (*p* < 0.001; Figure 1B). Figure 1C shows the dose-response curve of intrathecal OT-induced scratching behaviors. As an indication of drug potency, the EC_50_ of intrathecal OT-induced scratching was 0.0159 nmol/injection. These results suggested that OT is highly effective in triggering strong scratching behaviors in mice when it is given intrathecally. Intrathecal OT-induced scratching behavior showed no difference between mice of different sexes (*p* > 0.05, Figure 1D). In addition, pretreatment of morphine (0.3 nmol, i.t.) had no effect on OT-induced scratching (Figure 1I), indicating that OT-induced scratching is not a pain behavior.

### Spinal OTRs Mediated OT-Induced Scratching Behavior

Selective OTRs agonist, TGOT (0.03 nmol, i.t.) produced significant scratching behaviors, which were equivalent to OT (*p* > 0.05, Figure 1E). TC OT 39 (0.1 nmol, i.t.), which is not only a OTRs agonist, but also a vasopressin 1a receptor (V1aR) antagonist, also mimicked OT-induced scratching behavior (*p* > 0.05; Figure 1F). Blocking OTRs by pretreatment of its selective antagonist, Atosiban (0.1 nmol, i.t.) or dVOT (0.1 nmol, i.t.) significantly decreased 0.03 nmol of OT-induced scratching behaviors (*p* < 0.05; Figure 1G). Moreover, intrathecal injection of RG7314, a selective V1aR antagonist at dose of 0.1 nmol did not affect OT-induced scratching behaviors (*p* > 0.05; Figure 1H). These results suggested that intrathecal OT-induced scratching behavior is mediated by the spinal OTRs.

### Grp mRNA and Otr mRNA Were Co-expressed in the Superficial Dorsal Horn

**FIGURE 2:**
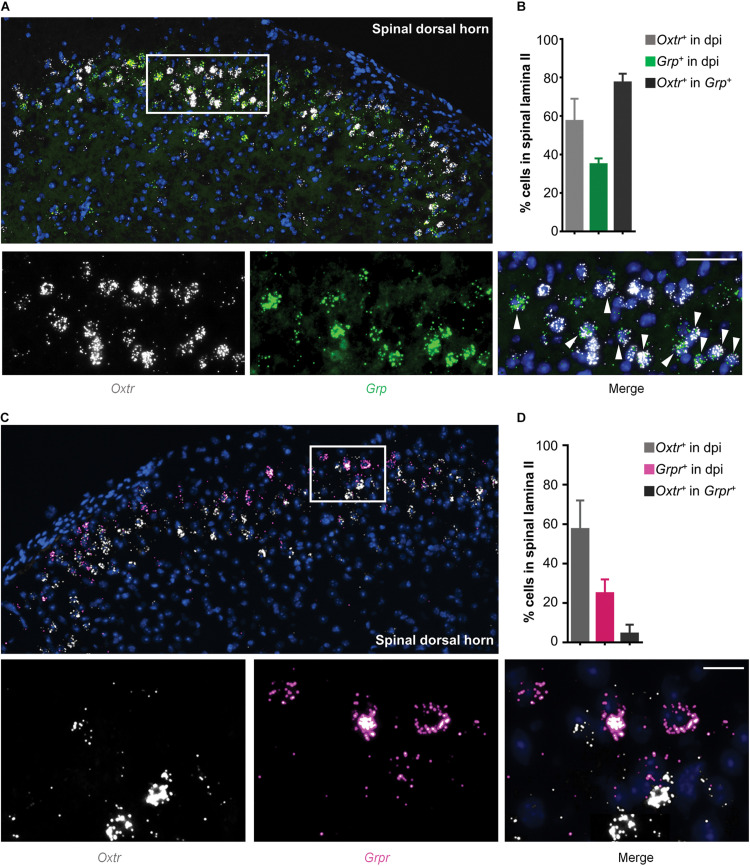
Co-expression of *Otr* mRNA and *Grp* mRNA in spinal dosal horn. **(A)** *In situ* hybridization for *Otr* and *Grp* mRNA on spinal dorsal horn sections. Co-expression of *Otr* mRNA (white) and *Grp* mRNA (green) in the enlarged image. Arrowheads indicate the co-expression of *Otr* and *Grp* mRNA. *n* = 9–15 sections from 3 mice. **(B)** percentage of neurons expressed *Otr, Grp*, or *Otr-Grp* mRNA. Data are expressed as mean ± SEM. **(C)** *In situ* hybridization for *Otr* and *Grpr* mRNA on spinal dorsal horn sections. Co-expression of *Otr* mRNA (white) and *Grpr* mRNA (pink) in the enlarged image. Arrowheads indicate the co-expression of *Otr* and *Grpr* mRNA. *n* = 9–15 sections from 3 mice. **(D)** percentage of neurons expressed *Otr, Grpr*, or *Otr-Grpr* mRNA. Data are expressed as mean ± SEM.

Since GRP and GRPR neurons are important components of itch transmission in the superficial dorsal horn, we used *in situ* hybridization assay (RNAscope) to detect the properties of *Otr* mRNA and *Grp/Grpr* mRNA distributions in the superficial dorsal horn. As shown in Figure 2A, most *Otr* (white) and *Grp* (green) mRNA positive neurons were concentrated in the superficial dorsal horn, and near 80% (4 mice) *Grp* mRNA positive neurons expressed *Otr* mRNA (Figures 2A,B). In contrast, few *Grpr* mRNA positive neurons (near 5%, 4 mice) expressed *Otr* mRNA (Figures 2C,D). These findings implied that OTRs are mainly expressed in GRP, but not GRPR neurons, in the superficial dorsal horn.

### Ablation of GRPR Positive Neurons Reduced OT-Induced Scratching Behaviors

**FIGURE 3:**
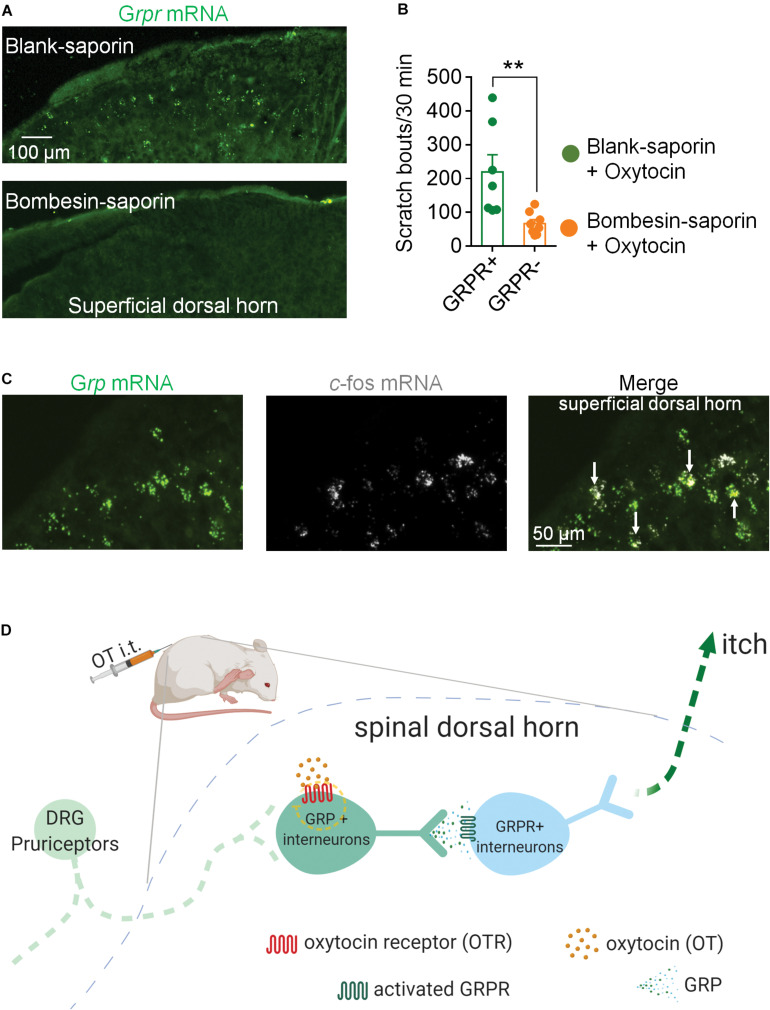
Ablation of GRPR neurons reduced OT-induced scratching behaviors in mice. **(A)** RNAscope showed Bombesin-saporin selectively ablated spinal GRP neurons. **(B)** Ablation of GRPR neurons reduced OT-induced scratching behaviors in mice. Mice were given a single intrathecal injection of Bombesin-saporin (400 ng). Intrathal OT-induced scratching bouts was then recorded for 30 min two weeks after Bombesin-saporin injection. Data are expressed as mean ± SEM. Student *T*- test. **(C)** Co-expression of *Grp* mRNA and *c-fos* mRNA in spinal dorsal horn. *In situ* hybridization for *Grp* (green) and *c-fos* (white) mRNA on spinal dorsal horn sections. Arrows indicate the co-expression of *Grp* and *c-fos* mRNA. *n* = 9–15 sections from 3 mice. **(D)** Schematic illustration of proposed spinal neural circuit that transmit OT-induced itch scratching behavior in mice.

GRP neurons are relayed by GRPR neurons in the spinal itch pathway. Next, we ablated GRPR neurons by a single intrathecal injection of Bombesin-saporin (400 ng). We found that spinal *Grpr* mRNA was hard to be detected 2 weeks after the injection of Bombesin-saporin (Figure 3A). Meanwhile chemical ablation of GRPR neurons significantly reduced intrathecal OT-induced scratching in mice. The scratching bouts in 30 min after OT injection decreased by 70% in the Bombesin-saporin group compared with the control (*p* < 0.01; Figure 3B). This result demonstrated that GRPR neurons are transmission neurons of intrathecal OT-induced itch scratching behaviors.

### OT Activates GRP Neurons in the Superficial Dorsal Horn

To test whether OT activates GRP neurons in the spinal cord, we performed RNAScope to show the localization of *c-fos* mRNA and *Grp* mRNA in the superficial dorsal horn 20 min after intrathecal injection of OT. As the arrows indicated in Figure 3C, some *Grp* mRNA positive neurons also expressed *c-fos* mRNA, suggesting that these GRP neurons are activated by OT injection.

### Intradermal Injection of OT Failed to Evoke Scratching Behaviors in Mice

**FIGURE 4:**
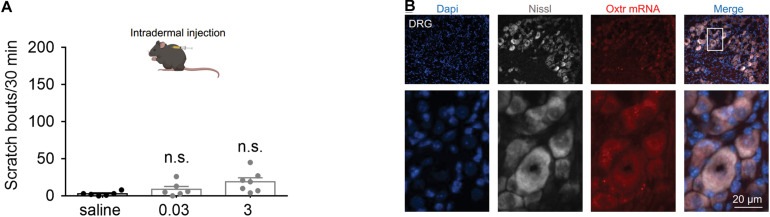
Systemic OT failed to evoke scratching behaviors in mice. **(A)** Intradermal injection of OT failed to evoke scratching behaviors in mice. OT was administered intradermally, scratching bouts were then recorded for 30 min. Data are expressed as mean ± SEM. Student *t*-test. **(B)** The nuclei (blue) were counterstained with Dapi, and cell body of the neurons (white) was shown by Nissl staining. RNAScope data showed that *Otr* mRNA (red puncta) was not expressed in the DRG neurons. *N* = 9–15 sections from 3 mice. Scale bar, 100 μm.

To determine whether peripheral administration of OT evokes scratching behavior, the effect of intradermal injection of OT was tested. We found that intradermal injection of OT (0.03 and 3 nmol) failed to evoke significant hindpaw scratching behaviors in mice (Figure 4A). In Figure 4B, the nuclei (blue) were counterstained with Dapi, and cell body of the neurons (white) was shown by Nissl staining. RNAScope data revealed that *Otr* mRNA (red puncta) was not observed in the DRG neurons (Figure 4B). Since more than 5 puncta per cell are classified as positive (Wang et al., 2020), this result suggested that OTR are not expressed in the DRG neurons. This might be the reason why peripheral OT does not cause itch scratching behaviors in mice.

## Discussion

In the present study, we first showed that intrathecal OT, within a dose range of 0.01 to 0.3 nmol induced robust but transient hindpaw scratching behavior in mice. We found that pharmacological activation of OTRs caused similar scratching behavior as OT. Meanwhile, blocking spinal OTRs, but not V1aR, significantly reduced this behavior, suggesting spinal OTRs as the primary receptor to mediate OT-induced scratching behaviors. Further RNAscope studies revealed that near 80% spinal GRP neurons expressed OTRs. OT activated the expression of *c-fos* mRNA in spinal GRP neurons. Chemical ablation of GRPR neurons significantly reduced intrathecal OT-induced scratching behaviors, suggesting GRP/GRPR neurons as the spinal transmission neurons for OT-induced scratching behavior. Finally, we also demonstrated that peripheral administration of OT failed to cause scratching behavior in mice, which may be due to the lack of OTRs expressed in DRG neurons. To our knowledge, this is the first study to demonstrate that GRP/GRPR pathway mediates intrathecal OT-induced scratching, and suggested this scratching as an itch behavior.

While studying the analgesic effect of OT, we observed that intrathecal OT caused significant and robust hindpaw scratching behavior in mice and rats. This scratching behavior occurred in bouts beginning immediately after injection and lasted about 30 min. This intense and acute scratching behavior is usually masked by the analgesic effect of OT. However, the starting and peak time of these two actions are different: intrathecal OT-induced hindpaw scratching behavior is most obvious within 5 min, whereas the analgesic effect of intrathecal OT is most significant during 20–30 min after injection (Sun et al., 2018). These findings caught our attention. We consulted the relevant literatures and found that it has been well reported that central administration of OT elicits strong scratching and grooming behaviors in rodents (Meisenberg and Simmons, 1982; Van Wimersma Greidanus et al., 1990; Thurston, 1992; Schorscher-Petcu et al., 2010; Yaksh et al., 2014). For example, intracerebroventricular injection of a high dose (1 nmol) of OT caused behavioral symptoms such as grooming, moving, foraging and squeaking (Delanoy et al., 1978). In another study, intracerebroventricular injection of OT evoked scratching as the predominant symptom. Other behaviors including squeaking, foraging through wood shavings, occasional jumpings and head-to-tail circling were also observed in the study (Meisenberg and Simmons, 1982). The dose of OT needed to cause these effects was 1 to 30 nmol, except scratching, which usually occurred following 0.005 to 0.02 nmol OT administration (Meisenberg and Simmons, 1982). These results suggested that a relatively low dose of OT produces scratching rather than grooming in mice. However, OT-induced scratching behavior is usually observed when studying the analgesia or other effects of OT, and often regarded as toxic side effects or simple pharmacological consequences of OT. Due to its short duration or anesthetic operation, this scratching behavior is easily overlooked or masked. Few studies have investigated this behavior and its neural mechanisms. Since this scratching behavior is widely reported without in-depth research, it deserves further investigation. Understanding the mechanisms of OT-induced behavioral change will be important in further clarifying how OT controls behavior and cognition, and for the development of OT-based drugs to treat a variety of psychiatric disorders.

In the present study, we observed the scratching behaviors induced by different doses of OT, which showed the following features: firstly, intrathecal OT was highly effective in triggering robust hindpaw scratching behavior in mice with its EC_50_ being 0.0159 nmol under our experimental conditions. Secondly, OT induced scratching behavior occurred very quickly after injection. It was strong but transient with the most scratching happened within 5 min after injection. One explanation for the short duration of OT-induced scratching would be the rapid decrease of OT concentration at the synapse with its primary receptor. Indeed, the effect of OT on behavioral arousal was enhanced by amastatin, a drug that inhibits several aminopeptidases in the central nervous system (Meisenberg and Simmons, 1984). Thirdly, within a dose range of 0.01 to 0.1 nmol, OT produced scratching rather than grooming as the predominant symptom of behaviors. This finding was consistent with the previous report that a low dose of OT produced scratching, whereas OT at a dose 10 times higher produced fore limb behaviors as grooming (Meisenberg and Simmons, 1982). It is likely these two kinds of behaviors are mediated by different neural mechanisms. Previous study showed that the PVN of the hypothalamus appears to be important in the integration of grooming, but not scratching (Eguibar et al., 2015).

Since OT plays its effects mainly by activating OTRs in the spinal cord (Jiang et al., 2014; Sun et al., 2018), we then confirmed whether spinal OTRs mediate intrathecal OT induced scratching behavior. Selective activating OTRs by its agonist, TGOT or TC OT39 (also an antagonist of V1aR) produced scratching behaviors equivalent to that induced by the same dose of OT, whereas antagonizing OTR by its antagonist, Atosiban attenuated OT-induced scratching behaviors, indicating that OTRs mediates intrathecal OT induced scratching behavior. In 2010, Schorscher-Petcu et al described in their study that V1aR, but not OTR, mediated OT-induced scratching behavior (Schorscher-Petcu et al., 2010). The inconsistence may be explained by different doses and sites of injection of OT. We used 0.03 nmol of intrathecal OT, whereas Schorscher-Petcu et al used 0.1 nmol of i.c,v. OT. As the dose of OT increases, its affinity for other receptors, such as V1aR and transient receptor potential vanilloid type-1 (TRPV1) may increase (Kubo et al., 2017; Nersesyan et al., 2017). Although OT and AVP and their receptors have very similar chemical structures, their actions are different or even opposite (Legros, 2001; Heinrichs et al., 2009; Neumann and Landgraf, 2012; Stoop, 2012). Although 0.1 nmol of V1aR antagonist RG7314 did not affect OT-induced scratching, we cannot rule out the involvement of V1aR, because only a single dose of RG7314 was included, and we did not test the effect of RG7314 on higher doses of OT-induced scratching. Taken together, these results suggested that spinal OTRs mediate OT-induced scratching behavior.

It is noteworthy that blocking OTRs did not completely abolish OT-induced scratching behavior in this study. Other mechanisms exist to regulate OT-induced scratching behavior. OT may evoke scratching by directly acting on the central pattern generator of scratching, in which the motor neurons receive synapses from descending oxytocinergic pathways and then activate the rhythmic pattern (Spruijt et al., 1988). Dopaminergic pathways have also been suggested to mediate OT-induced grooming and scratching. Intra-accumbens administration of haloperidol, a dopaminergic antagonist, suppressed OT induced grooming and scratching (Drago et al., 1986).

Hindpaw scratching is a characteristic itch behavior of rodents, in which the animals use the hind limb on the same side to scratch. It is distinguished from noxious stimuli evoked fore limb wiping or head-shaking response (Pan et al., 2019). Recently, Li et al. reported that intradermal injection of OT did not elicit itch scratching, but aggravated chloroquine-induced itch responses in mice (Li et al., 2019). Clinical evidence showed that patients who received OT showed a risk of experiencing pruritus (Boucher et al., 2004; Rath, 2009). Moreover, OT-induced grooming and scratching behavior can be blocked by an opiate receptor antagonist, naloxone (Van Wimersma Greidanus et al., 1990). Opioid receptor antagonists was also shown to reduce scratching following dermatitis, uremic pruritus, and anti-PD1 immunotherapy (Brune et al., 2004; Kwatra et al., 2018; Reszke and Szepietowski, 2018; Serrano et al., 2018; Singh et al., 2019). This reduction in scratching is thought to reflect a relief of itch sensation, because opiate antagonists reduced experimental itch sensation in humans (Heyer et al., 1997). Taken together, we hypothesized that OT induced scratching is an itch symptom.

To further investigate the underlying spinal transmission neurons of OT-induced scratching, we performed RNAScope to investigate the distribution properties of *Otr* and *Grp/Grpr* mRNA in the spinal cord. We observed a high co-expression of *Otr* and *Grp* mRNA. In contrast, few *Grpr* mRNA positive neurons expressed *Otr* mRNA. OT activated the expression of *c-fos* mRNA in spinal GRP neurons. Chemical ablation of spinal GRPR neurons significantly reduced intrathecal OT-induced scratching behavior. These results suggested that GRP/GRPR neurons appear to serve an important role in OT-induced scratching behavior. Considering OTRs are expressed in GRP neurons, but not GRPR neurons, it is possible that OT first binds to the OTRs expressed on GRP neurons to release GRP, which then activates GRPR neurons to trigger scratching behavior in mice (Figure 3C).

OTRs belong to the G protein-coupled receptors superfamily, specifically coupled to the Gq/11 a-class guanosine triphosphate (GTP) binding proteins. The Gq/phospholipase C (PLC)/inositol 1,4,5-triphosphate (InsP3) pathway is the major pathway mediating the signal of OTR after binding of OT to its receptor (Gimpl and Fahrenholz, 2001; Smith, 2007). Based on our results, it is possible that OT binds to OTRs expressed on the GRP neurons and through second messengers PLC/InsP3 to release Ca^2+^ ions from intracellular stores. Intracellular Ca^2+^ ions then activate GRP neurons and release GRP.

We also showed in the present study that intradermal injection of OT failed to evoke hindpaw scratching behaviors in mice. Itch signal from the periphery tissue to the spinal cord is conducted by DRG (Dong and Dong, 2018). These neurons synapse with second-order neurons in the spinal dorsal horn for further processing. RNAscope experiment demonstrated that DRGs neurons expressed little OTRs, which may explain why peripheral OT does not cause itch scratching behavior. This may also be the reason why itch is not a notable side effect of OT in human, most of clinical OT adopts systemic administration.

## Conclusion

In conclusion, we used behavioral and pharmacological tests, RNAscope and chemical ablation to demonstrate that intrathecal OT binds to the OTRs expressed on GRP neurons, and activates GRP/GRPR pathway to trigger itch-scratching behavior in mice. These findings provide novel evidence relevant for advancing understanding of OT-induced behavioral changes, which will be important for the development of OT-based drugs to treat a variety of psychiatric disorders.

## Data Availability Statement

The raw data supporting the conclusions of this article will be made available by the authors, without undue reservation.

## Ethics Statement

The animal study was reviewed and approved by the Animal Care and Use Committee of Health Science Center at Shenzhen University.

## Author Contributions

JG, XB, and PW: investigation, methodology, validation, formal analysis. MM, WS, LX, DX, and XL: investigation, methodology. CJ: conceptualization, data curation, funding acquisition, resources, writing – review and editing, supervision. YH: conceptualization, data curation, writing – original draft, writing – review and editing, supervision, project administration, funding acquisition.

## Conflict of Interest

The authors declare that the research was conducted in the absence of any commercial or financial relationships that could be construed as a potential conflict of interest.
